# Approaches to Design an Efficient, Predictable Global Post-approval Change Management System that Facilitates Continual Improvement and Drug Product Availability

**DOI:** 10.1007/s43441-024-00614-9

**Published:** 2024-02-18

**Authors:** Anders Vinther, Emma Ramnarine, Thierry Gastineau, Laura O’Brien, Oliver Brehm, David Fryrear

**Affiliations:** 1 QBA – Quality Business Administration, San Carlos, CA USA; 2 Boehringer Ingelheim, Fremont, CA USA; 3 Sanofi, Paris, France; 4 CSL Behring, King of Prussia, PA USA; 5 Bayer, Berlin, Germany; 6 Astellas, Northbrook, IL USA

**Keywords:** Post-approval changes, Quality management, Regulatory complexity, One-voice-of-quality for post-approval changes (1VQ for PAC), Drug shortages

## Abstract

The complexity and inter-connectedness of operating in a global world for drug product supply has become an undeniable reality, further underscored by the COVID-19 pandemic. For Post-Approval Changes (PACs) that are an inevitable part of a product’s commercial life, the impact of the growing global regulatory complexity and related drug shortages has brought the *Global* *PAC* *Management* *System* to an inflection point in particular for companies that have their products marketed in many countries.

This paper illustrates through data analyzed for the first time from 145,000 + PACs for 156 countries, collected by 18 global pharma companies over a 3-year period (2019–2021), how severe the problem of global regulatory complexity is. Only PACs requiring national regulatory agency (NRA) approval prior to implementation were included in the data set. 1 of the 156 country NRAs approved all submitted PACs within a period of 6 months. The 6-month timeline was chosen because it is the recommended review timeline for major changes in the WHO guidance for vaccines and biotherapeutic products. 10 out of the 156 (6%) countries had no more than 10% of the PACs reviewed and approved in > 6 months. In 33 (22%) countries more than half of the PACs took > 6 months for approval. It is rare that the same PAC is approved globally within 6 months as individual NRAs take from a few months to years (in some cases > 5 years) for their review.

The global PAC management complexity has steadily grown over the past 20 years. Attempts thus far to solve this problem have not made any meaningful difference. Senior leaders and decision-makers across the interdependent components of the complex Global PAC Management System (industry and regulators) must come together and collaboratively manage the problem holistically with the objective of ensuring global drug product availability instead of continuing with distinct stakeholder or country-focused solutions, which can tend to worsen the problem.

In this paper, the Chief Quality Officers (CQOs) from 18 of the largest innovator pharma companies (see Acknowledgements) are speaking with One-Voice-of-Quality for PACs *(1VQ for PACs Initiative)*. They are recommending a set of 8 approaches to activate a holistic transformation of the Global PAC Management System. This article presents their view on the problem of global regulatory complexity for managing PACs, it’s impact on continual improvement and the risk to drug product supply, as well as approaches that can help alleviate the problem.

## Introduction

Changes are inevitable during the commercial life of a product and continual improvement is an expectation for product lifecycle management. ICH Q10 defines continual improvement as *a recurring activity to increase the ability to fulfill requirements* [1, 2, 3]. Changes are needed for instance, to upgrade (aging) manufacturing and testing facilities and equipment, close cGMP compliance gaps, change suppliers, implement new regulatory requirements, new technologies, and emerging new knowledge, to continually improve products and processes. These changes are called Post-Approval Changes (PAC) as they occur after the initial approval of a new drug product. (Note: PACs might be referred to by other terminology in different countries, e.g., variations in the European Union. In this paper the term PAC refers only to changes to the Chemistry, Manufacturing and Control (CMC) section (Module 3) of the Common Technical Document (CTD), that are introduced after the first approval of the drug product in a country). PACs need to be managed along the product lifecycle, which may last over several decades.

Many PACs require prior approval by individual national regulatory agencies (NRAs) where the product is marketed. Each PAC is first assessed and internally approved by the company’s Quality Assurance department and documented in the company’s Pharmaceutical Quality System (PQS). Only PACs that meet cGMPs, company and NRA requirements should be internally approved. The PAC is then submitted for regulatory approval to each NRA where prior approval is required. For a globally registered product this may mean several dozen individual submissions for the same PAC. Each NRA has their own documentation requirements, thus the company typically prepares several different submission packages for the same PAC. The NRA conducts a science and cGMP compliance assessment and decides on regulatory approval or rejection of the PAC. Companies must balance anticipated supply need for each product version with the estimated approval timeline by each NRA for each PAC. NRAs do not commit to a particular approval timeline and as such, full global approval of a PAC (from all NRAs that the PAC was submitted to for prior approval) can usually take 3–5 years [4]. What further complicates matters is that the same PAC is often not submitted to all NRAs at the same time. Different companies might have different PAC submission strategies. Also, some NRAs act as reference countries for other NRAs meaning that PAC approval by the reference NRA is required before the company can submit the same PAC to other NRAs relying on the reference country. Large pharmaceutical companies with products marketed worldwide have several thousand prior approval PAC submissions per year. Generally, WHO maturity level 3 (ML3) and 4 (ML4) countries are better at reviewing PACs in line with WHO timeline recommendations than ML1 and ML2 NRAs. Companies must consider how many parallel versions of a product it can manage simultaneously. One of the authors’ own experience is that in one year 83 batches of a vaccine product were produced according to 55 different variations. This puts a tremendous challenge on managing inventories of the many versions. Thus, some companies simply wait until a certain number of countries have approved the PAC before implementing it. In a perfect world, companies would submit the PAC to all affected NRAs simultaneously and all would review the PAC within the 6 months suggested by WHO.

The global complexity for PACs arises because of national differences in regulatory processes, reporting levels and requirements, approval timelines, and overall redundant scientific assessments of each PAC by many NRAs. The complexity and prolonged approval timelines disincentivize companies to invest in continual improvement projects and makes it challenging for NRAs to review the huge volume of PACs.

There is a general recognition of the global regulatory complexity problem for PACs. Attempts have been made to solve the complex problem for over 20 + years; yet no substantial progress has been made. This paper contends that the reason for no meaningful impact thus far, is because the problem has not been explored as a “complex problem” with the objective to improve the **Global** *PAC Management System* (also referred to as ‘the system’ in this paper) as a whole. Attempted solutions have taken a linear cause-and-effect approach which might work for complicated problems, but not for complex problems. Roberto Poli (University of Trento, Italy) provides the following distinction between the two [5]:

Complicated problems originate from causes that can be individually distinguished; they can be addressed piece by piece; for each input to the system there is a proportionate output; the relevant systems can be controlled and the problems they present admit permanent solutions.

On the other hand, complex problems and systems result from networks of multiple interacting causes that cannot be individually distinguished; must be addressed as entire systems, that is they cannot be addressed in a piecemeal way; they are such that small inputs may result in disproportionate effects; the problems they present cannot be solved once and for ever, but require to be systematically managed and typically any intervention merges into new problems as a result of the interventions dealing with them.

When a complex problem is misdiagnosed as a complicated problem, individual attempts by a stakeholder, while reasonable and well-intended, can often make the problem worse.

William Edwards Deming, known as a leading thinker for Quality Management^1^ demonstrated that companies which apply a ‘systems approach’ outperform those that don’t. Deming defined a system as "a network of interdependent components that work together to accomplish the aim of the system". He further stated that "Management of a system requires knowledge of interrelationships between all of the components within the system and of everybody that works in it"*.* [6]. His System of Profound Knowledge includes a) theory of knowledge, b) knowledge of variation, c) appreciation for a system, and d) psychology of change. The foundational concept is that if only one part of the system is optimized, or if each stakeholder independently optimizes only their part of the system, the overall system will continue to underperform and remain sub-optimal. Applying Poli’s concepts and Deming’s teachings, improvement of the Global PAC Management System will only be possible when stakeholders work together and are able to dynamically manage activities across the inter-related components of the system, i.e., the system design allows stakeholders to change, learn and adapt as new scenarios present [5].

An effective Global PAC Management System would be one where the system is jointly owned, collectively managed, and navigated in partnership by all stakeholders to continually improve and deliver high quality medicines on time every time. Today PACs are managed by NRAs with a country or regional level approach without any global coordination.

This paper is written and endorsed by the Chief Quality Officers (CQOs) from 18 of the largest innovator pharmaceutical companies (See Acknowledgements). The CQOs are accountable for their company’s Pharmaceutical Quality System (PQS), current Good Manufacturing Practice (cGMP) compliance, assessing suitability of changes, and releasing quality drug products to patients worldwide. These CQOs have come together to speak with One-Voice-of-Quality for Post Approval Changes (*1VQ for PAC Initiative)*. They have defined and published what an effective PQS is for managing PACs at a system and individual change level [7, 8].

In this paper, the CQOs present data that illustrates the magnitude of the complex PAC problem and recommend 8 approaches to alleviate the problem by activating a holistic way to navigate the complexity of the Global PAC Management System.

## Materials and Methods

This review article illustrates through the most comprehensive dataset presented to date the severity of the complex global PAC regulatory framework problem.

18 large global pharma companies gathered data for all prior-approval PACs over a period of 3 years (2019–2021). Included in the dataset are all PACs that were approved in the specific year (irrespective of when it was submitted to the NRA). The companies shared the number of PACs that took longer than 6 months for approval from date of submission to each NRA, and the number of PAC approvals that took no more than 6 months for approval for each of the years 2019, 2020, and 2021. 16 of the 18 companies provided additional approval detail to the specific country level for all PACs.

Some PACs require prior approval in some countries and not in others. PACs that do not require prior approval such as Changes Being Effected (CBE) and Annual Reportable (AR) were not included in the dataset as these can be implemented at a time decided by the company without the uncertainty of awaiting approval from relevant NRAs. Only prior approval PACs were included in the dataset as this is where the biggest challenge exists with efficient and predictable change planning and implementation.

The total number of PACs approved globally (146,550) was calculated by addition of data from all 18 companies over the 3 years. The percentage of PACs that were approved beyond 6 months since submission to each NRA was calculated.

Where data were provided at country level the total number of PACs for the specific country was calculated by addition of data from all companies. The percentage of PACs that was approved beyond 6 months was calculated. Data for 156 countries with more than 10 PACs over the three years were included in this article (125,886 PACs). All country names were anonymized in the figures and tables in this article.

The 6-month time period was chosen because it is the recommended review timeline in the WHO guidance for vaccines [1] and biotherapeutic product [2] for major changes (6 months for vaccines, 3–6 for biotherapeutic products). Moderate changes are recommended by WHO to be reviewed within 3 months for vaccines and 1–3 months for biotherapeutic products. We were unable to find recommended review timelines for small molecules.

It is important to distinguish between NRA review and approval times for a PAC. NRAs might have questions to the company, which could result in additional time required before final approval. However, for the company what really matters for change planning and implementation is the final approval (or rejection) of a PAC. In this paper we therefore chose the time period for approval and believe that all stakeholders should work towards no more than 6 months from original submission of the PAC to a final decision rendered (approval or rejection) by the NRA. Predictable and shortened overall global timelines for NRA PAC review is the number one improvement that would help reduce the supply chain complexity caused by the need to manage several product versions at the same time.

## Results

CQOs of the largest 25 pharma companies were requested to provide the following detailed PAC data for 2019, 2020 and 2021.

- How many prior-approval PACs were approved in $\le$ 6 months per country?
- How many prior-approval PACs were approved in $>$ 6 months per country?

18 out the 25 companies provided their PAC data. Detailed data per year and country were received from 16 companies for a total of 125,886 PACs. 2 additional companies provided data per year consolidated at the global level (i.e., not at country level) bringing the grand total number to 146,550 PACs across 156 countries.

**Figure 1:**
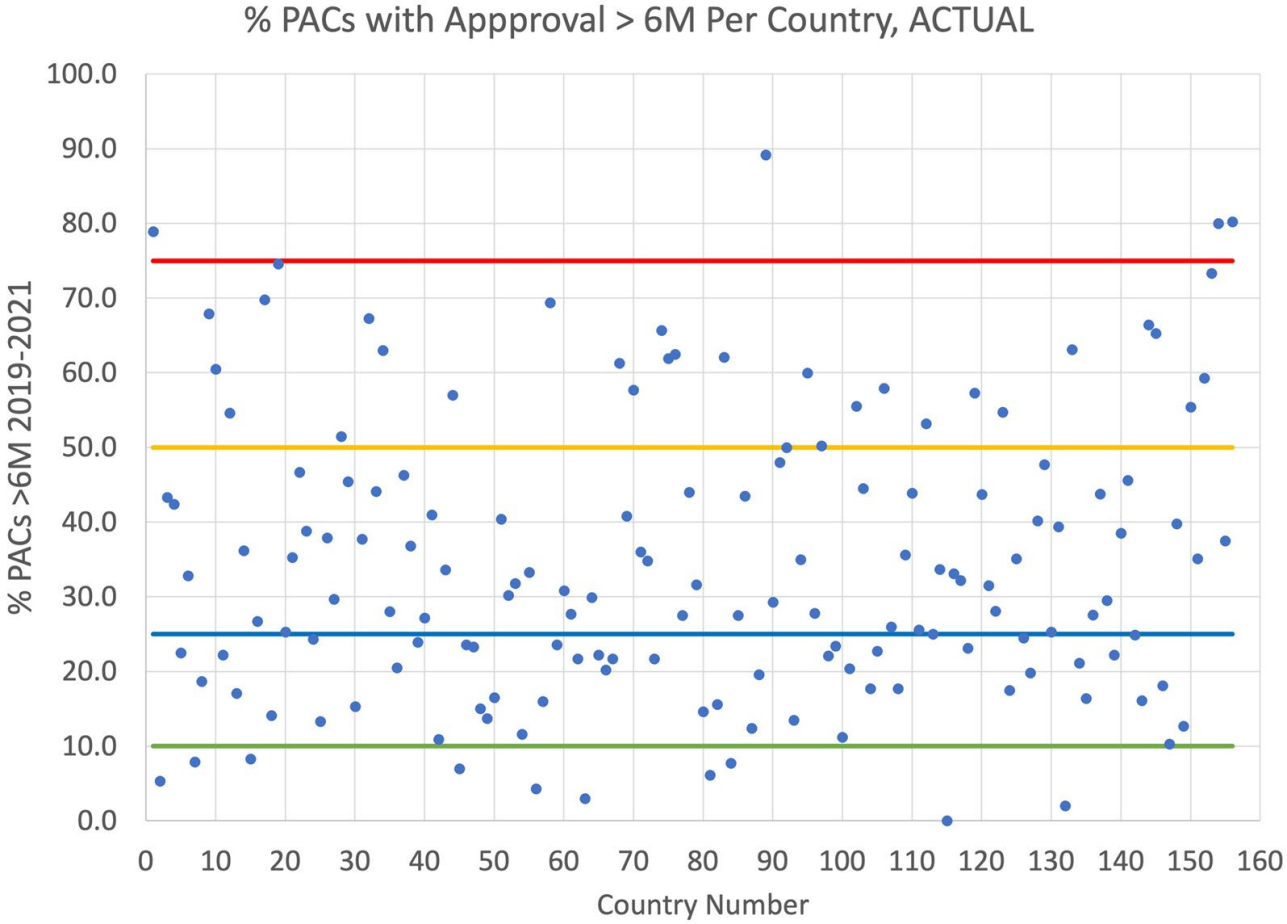
Percentage of PACs approved in > 6 M Per Country 2019–2021.

For each of the 156 countries the aggregate percentage of PACs (2019–2021) that were approved past 6 months (> 6 months) was calculated. In an ideal situation all PACs would be assessed and approved by the relevant NRAs within 6 months of submission. The actual data is shown in Fig. 1, with the X-axis being an assigned country number (country name anonymized) and the Y-axis being the *percentage of PACs* that took more than 6 months for approval. Each dot in the figure represents one country (NRA). What the figure shows is the percentage of prior approval PACs that took more than 6 months for each of the 156 countries. The figure does not show the total number of PAC that the NRA approved. It also does not show how long it took for a company to get each PAC approved by all countries (globally). (Note: The vast majority of prior approval PACs are approved without significant changes from the original submission. In an informal survey among major vaccine manufacturers > 99% of all prior approval PACs were approved).

Figure 1 data is tabulated in Table 1 below and shows that only one country (out of 156) approved all PACs in $\le$ 6 months. A PAC cannot be implemented until a significant number of countries have approved it. Thus, even though 10 countries (6%) approved at least 90% of PACs within 6 months the company submitting the PAC must manage several versions of the product at the same time because of the highly varying approval timelines *globally* for *each* PAC. This is a logistics challenge that can cause drug shortages. It is rare that any PAC is approved globally in less than 6 months.

**Table 1:** Distribution of Countries and PAC approvals taking > 6 M

Percentage (of 125,886) PACs taking > 6 months for approval	Number of Countries	Percentage (of 156) countries
0%	1	< 1%
1–10%	9	6%
10–25%	49	31%
25–50%	64	41%
50–75%	29	19%
75–100%	4	3%

The 2019 FDA Drug Shortages Report [9] stated that 62% of all drug shortages in the US, 2013–2017, were caused by manufacturing quality issues. Undoubtedly, the industry is responsible for ensuring that manufacturing and testing of products and their PQS are cGMP compliant and that processes are continually improved. It is a paradox that while continual improvement is desired by both industry and NRAs, timely implementation of new knowledge for continual improvement is impossible, with global approval timelines being years rather than weeks or months. This complexity disincentivizes companies to continually improve.

It would be useful to explore and assess why some NRAs take years while others take months or weeks to approve the same PAC. Is it a matter of resource limitation? Is it an inadequate understanding of the differences among NRA (country) review processes/requirements and how these might contribute to the overall complexity?

Table 2 below provides the absolute number of PACs approved in each of the three years and the combined total for the 3 years. There is no significant trend in number of PACs or percentage of PACs taking more than 6 months for approval over the three years.

**Table 2:** Total number of PACs approved per year

Year	Total number of PACs approved	Number of PACs approved $\le$ 6 months	Number of PACs approved $>$ 6 monthsb	% PACs approved > 6 months
2019	47,285	33,547	13,738	29
2020	50,552	36,967	13,585	27
2021	48,713	35,344	13,369	27
2019–2021 combined	146,550	105,858	40,692	28

The data from the 18 companies show that the total number of active global PACs at any given time is substantial. On average, each company had about 2,700 PAC submissions approved per year.

As seen from Table 2 approximately 28% of the 146,550 changes took more than 6 months for NRA approval. While 72% of the individual PACs were approved by individual NRAs within 6 months, that does not mean that 72% were approved by ALL affected NRAs globally in 6 months. That is where the problem fundamentally lies. As a hypothetical example, let’s assume that a PAC requires prior approval by 60 countries and that it is submitted to all NRAs at the same time. In this example let’s assume that 35 of the NRAs approve the PAC within 6 months, an additional 10 within a year, 8 more in year 2, and that the remaining 7 NRAs take up to 5 years for their approval of the PAC. For the company this means that it will need to operate with two versions of the same product (one pre-change, and one post-change) until the last NRA has approved the change, i.e., 5 years in this example. If the last 7 NRAs (7/60 = 12%) had reviewed the PAC within 2 years (and not 5 years), the company would only need to operate with two versions for 2 years instead of 5 years. Thus, even when one PAC might be approved in a few months by many NRAs, it is the ‘tail’ of the NRA countries that take years to approve the PAC, resulting in companies having to manage several versions of the same product at the same time. This is further complicated by the fact that the same change is often not submitted to all NRAs at the same time. For drug products supplied globally it is therefore, not uncommon to operate with dozens of versions at the same time due to multiple open ‘in-review’ PACs. This makes drug product supply logistically very challenging. It is rare that *any* PAC is approved globally by all relevant NRAs within 6 months.

**Figure 2:**
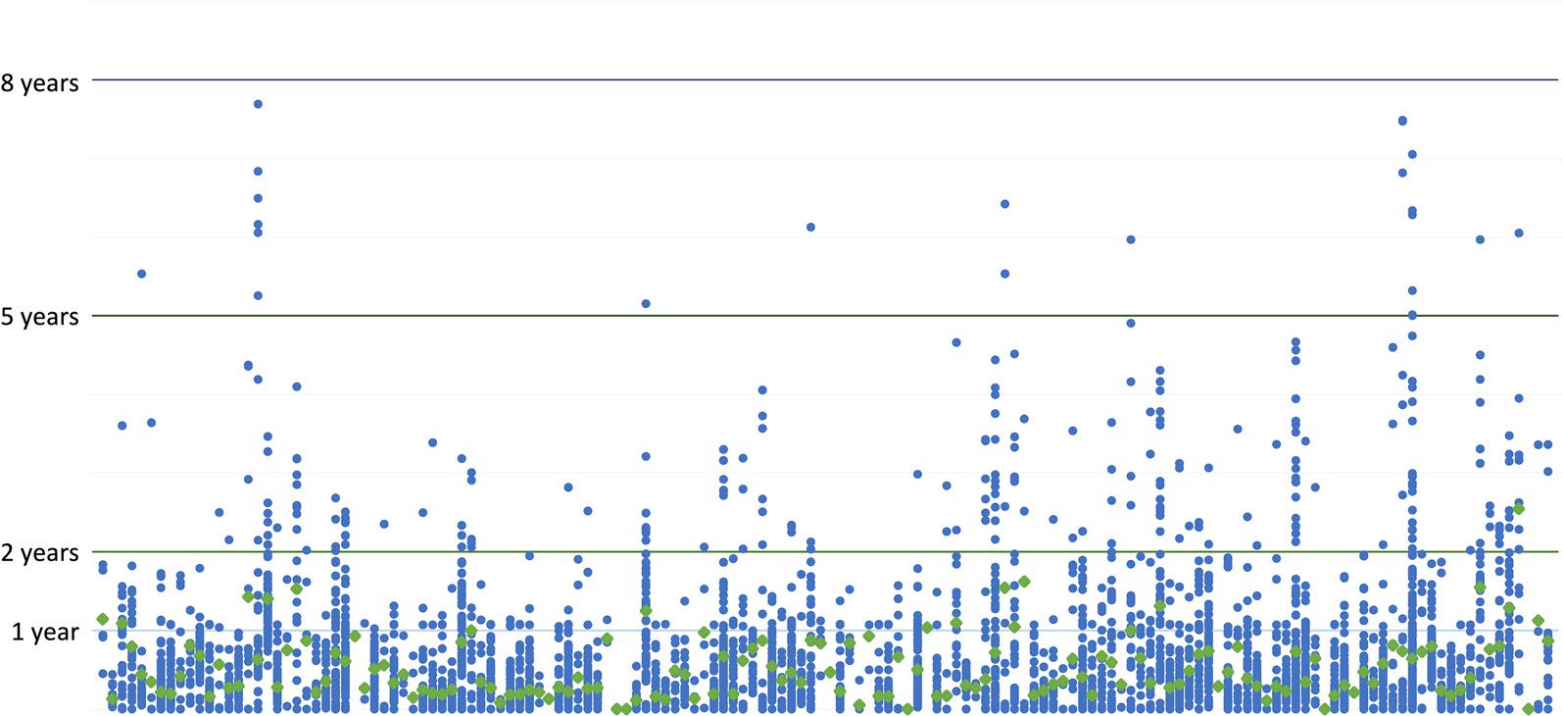
PACs and approval timelines data from one company.

Figure 2 below provides another angle to the PAC complexity. It shows 2019–2021 approved PAC data from one company showing the very large number of active PACs and approval times across all countries, 2019–2021. The X-axis represents individual countries, whereas the Y-axis in this figure shows *actual approval times* for individual PACs. Each blue data point is one approved PAC. The green dots represent the average time of approval for all PACs for each country. As seen, several PACs took more than 2 years to approve, with some taking almost 8 years.

It is evident from the data presented in this paper that the current global PAC management system is complex and simply not capable of agile handling of this astronomical volume of changes using current practices.

New knowledge should be implemented in a timely manner for continual improvement, but this is not possible in the current state. The overall Global PAC Management System needs to be transformed using systems thinking, to enable and incentivize a shift from a reactive to a proactive state, and advance agile continual improvement to reduce the risk of drug shortages for patients in all countries where a product is marketed.

## Discussion

### Global Recognition of the Problem and Its Consequences

The challenges with global regulatory complexity and its consequences have been known for over 20 years, as stated by Dr. Janet Woodcock in 2002 when she articulated a desired future state of “a maximally efficient, agile, flexible pharmaceutical sector that reliably produces high quality drugs without extensive regulatory oversight” [10]*.*

The various concepts and solutions suggested since then to achieve the desired state have stated the conditions and promise of regulatory flexibility. However, the laid-out conditions have mainly served to increase documentation and reporting requirements without any improvement in the regulatory flexibility for PACs, even when a science and risk-based approach clearly demonstrates that 1) a PAC does not increase risk to product quality and/or patient safety and, 2) regulatory flexibility to enable timely implementation via the PQS only, is warranted.

In 2005 a Concept Paper and Business Plan for ICH Q10, *Pharmaceutical Quality System* stated that “delays in the implementation of innovation and continual improvement for existing products may occur due to different expectations in the three regions” [11, 12]. Completed in 2008, ICH Q10 introduced new documentation requirements. It stated that companies demonstrating an effective PQS, and product and process understanding would have the opportunity to “optimize science and risk based post-approval change processes to maximize benefits from innovation and continual improvement” [3]*.* In other words, manage more PACs in the company’s PQS only without requiring prior-approval submissions.

The expected regulatory flexibility did not materialize as stated in 2014 in the ICH Q12, *Pharmaceutical Lifecycle, Concept Paper*, “The envisioned post-approval ‘operational flexibility’ (anticipated in ICH Q10) has not been achieved” [13]*.* ICH Q12 completed in 2019 states that the guideline “should enhance industry’s ability to manage many CMC changes effectively under the company’s Pharmaceutical Quality System (PQS) with less need for extensive regulatory oversight prior to implementation” [14]. The ICH Q12 guideline further introduced additional documentation requirements for PACs.

ICH Q9, *Quality Risk Management* [15], published in 2005, provided a science and risk-based application framework that would enable good product and process understanding, in conjunction with ICH Q8 (R2), *Pharmaceutical Development* [16], and timely decision-making by both industry and regulators. ICH Q10, Q9, Q8 were conceptually well-linked together, yet limited value has been realized from them in almost two decades. Science and risk-based decision-making has not improved as expected, as also confirmed in the 2020 ICH Q9 (R1) Business Plan—“the benefits of QRM, as envisaged by ICH Q9, have not yet been fully realised and this has also limited the value-realization of the other ICH Guidelines, such as ICH Q8 and Q10, which expect science- and risk-based approaches” [17].

The awareness of and discussions on drug shortages and PAC management have undoubtedly increased since the COVID-19 pandemic. The 2022 European Commission document, *Vulnerabilities of the Global Supply Chains of Medicines* states that “Industry representatives highlighted that the lack of international regulatory convergence combined with the complexity of legal frameworks across jurisdictions globally negatively affects the ability to respond in a flexible, effective, and timely manner to supply chain challenges” [18].

Attempting to move towards a more proactive state, in 2013 the US FDA initiated a Quality Metrics program with their latest recommendation on quality metrics published in 2022 [19]. The objective of the program includes proactively identifying and mitigating quality risks.

In 2022 FDA launched an additional Quality Management Maturity (QMM) Program. It proposes a QMM rating system which could “support increased flexibility for manufacturers to make post approval manufacturing changes with less regulatory oversight, incentivizing continual improvement” [20].

In 2021 PIC/S published a Recommendation Paper, *How to Evaluate and Demonstrate Effectiveness of the Pharmaceutical Quality System in Relation to Risk-Based Change Management* [21].

The International Coalition of Medicines Regulatory Authorities (ICMRA) in 2022 published a Pharmaceutical Quality Knowledge Management System (PQKMS) Reflection Paper that states, “ICMRA recognizes that pharmaceutical manufacturers seek agility to maintain robust supply chains and continually update manufacturing processes to incorporate changes and improvements as equipment ages, suppliers change, innovations are developed, and knowledge is gained. Companies manage these changes within their pharmaceutical quality systems and/or seek timely regulatory review when changes require prior approval. As the pharmaceutical industry is highly regulated, and the industry is globalized serving multiple markets, companies often must obtain these approvals from multiple national regulatory bodies with different timeframes, therefore potentially delaying implementation of changes” [22].

The common red thread across all these solutions so far, is that they have treated the global PAC complexity as a complicated problem and not as a complex problem. To alleviate the problem systems thinking must be applied to improve the global PAC Management System.

### Approaches Targeting the Design of an Efficient, Predictable Global PAC Management System

Achieving the desired future state of “a maximally efficient, agile, flexible pharmaceutical sector that reliably produces high quality drugs without extensive regulatory oversight” requires regulatory flexibility for managing PACs as outlined in ICH Q10, Annex 1 and ICH Q12. Without timely global approval of PACs within 6 months per the WHO guidelines [1, 2], and eliminating redundant reviews by each country, PACs cannot be implemented in a timely manner, and the current situation will not improve.

### What’s Being Done to Improve the Global PAC Management System Today

As Louis Pasteur said, “science knows no country, because knowledge belongs to humanity, and is the torch which illuminates the world”*.* Diseases also know no country. Pharmaceutical supply chains are global as well with respect to sourcing of raw materials and active ingredients, and distribution of final drug products. The overall management of PACs needs to be optimized at a global system level; approaches at a national, regional, or targeting a subset of countries, will not solve this global problem.

The 4 approaches below are in progress and have a common design theme of addressing processes across different stakeholder boundaries.

- Approach 1 (1VQ for PAC): How industry can accelerate manufacturing and quality improvements through a risk-based approach and an effective PQS—In 2020 the Industry *1VQ for PAC Initiative*, sponsored by the CQOs, published the paper, *Effective Management of Post-Approval Changes in the Pharmaceutical Quality System (PQS)—Through Enhanced Science and Risk- Based Approaches: Industry One-Voice-of-Quality (1VQ) Solutions* [7], that provides a standard step-wise risk-based process and decision tree solution to assess each PAC, and an approach to establish and demonstrate an effective PQS for PACs to “optimize science and risk-based PAC processes to maximise benefits from innovation and continual improvement” as envisioned by ICH Q10 Annex 1. The 1VQ for PAC Initiative is advocating that when companies demonstrate an effective PQS for managing PACs and have good product and process understanding they should be given opportunities to manage low risk changes in the PQS only, thus not requiring regulatory agency prior approval per ICH Q10. Such changes deemed as ‘do and tell’ could still be reported as Changes Being Effected (CBE) or in the Annual Report. The *1VQ for PAC Initiative* published an additional solution, *Industry One-Voice-Of-Quality (1VQ) Solutions Management Review (MR) of Post Approval Changes (PAC) Guide* [8], that provides practical guidance and examples of PQS Key Performance Indicators (KPIs) for PACs. When implementing these KPIs the company should be able to demonstrate how well they manage PACs in their PQS and discuss during Management Review the performance improvements (including efficiency and predictability improvements for PACs as a result of down-classification from prior-approval to a notification or managed only in the PQS).
- Approach 2 (PIC/S): How inspectors can evaluate, and companies can demonstrate an effective change management system—In 2021, PIC/S published a Recommendation Paper, *How to Evaluate and Demonstrate Effectiveness of the Pharmaceutical Quality System in Relation to Risk-Based Change Management* [21], providing a practical checklist for all steps of the change management process that when implemented would provide evidence of an effective change management system. It states, “It is considered that application by a pharmaceutical manufacturer of the guidance will provide evidence of the effectiveness of their PQS in relation to risk-based change management*.*” This single checklist can be used both by inspectors to evaluate the effectiveness of a company’s change management system, *AND* by companies to demonstrate that they can effectively manage PACs in their PQS. The intent, as stated in ICH Q10, Annex 1 and the PIC/S Recommendation Paper, is that companies demonstrating an effective PQS for managing PACs should be given more regulatory flexibility than those that don’t. PIC/S could provide a statement for companies or sites meeting the requirements for demonstrating an effective PQS for managing changes. This statement could be used by companies to request regulatory flexibility for PACs.
- Approach 3 (WHO): Increased reliance among regulatory agencies. WHO’s regulatory reliance guidance [23] encourages all NRAs to rely on the assessment and approval granted by a “WHO Listed Agency”, thereby enabling the acceleration of review and approval at the local NRA level. This guidance when used can significantly reduce the number of redundant assessments for PACs.
- Approach 4 (ICMRA): Pharmaceutical Quality Knowledge Management System (PQKMS) for PAC assessments—In 2021, ICMRA launched a PQKMS Initiative to provide global leadership for PAC assessments [22]. The objective is to accomplish this through harmonized, structured, and standardized data elements and electronic formats to enable simultaneous submissions to all associated regulatory authorities, secure sharing of information among multiple regulatory authorities, and more extensive reliance (most likely among higher resourced NRAs with adequate IT resources)

### What Still Needs to be Done?

The CQOs through this paper, are advocating for the following additional approaches for further design optimization of the Global PAC Management System; several of these are also discussed in a joint position paper from EFPIA, IFPMA and Vaccines Europe [24].

- New Approach 5: Industry and regulatory agencies jointly standardize and bring transparency to the process and data for assessing a PAC, based on the scientific/technical risk-basis for the change. The standard transparent data requirements and assessments, no matter which country, would result in a step change in efficiency and agility in PAC management. This approach would ultimately use the same template/process for assessing PACs by both the company and the NRAs.
- New Approach 6: Adoption of the WHO guidance on PAC review timeline of 6 months and the WHO guidance on regulatory reliance by all NRAs. All NRAs should adopt and consistently implement into national regulation the WHO recommended maximum PAC review timelines—maximum 6 months for major changes and a maximum of 3 months for moderate changes [1, 2]. This along with WHO’s regulatory reliance guidance [23], will reduce the number of redundant assessments of PACs.
- New Approach 7: A consistent approach for how assessors should consider PQS effectiveness assessment by inspectors, when deciding on PAC reporting levels. This would help assessors decide better on the reporting level for a change depending on how effective a company’s PQS is in managing PACs. It would also allow for consistency and alignment in the use of an effective PQS to downgrade PAC reporting levels.
- New Approach 8: All stakeholders should set up metrics and regularly publish data on PAC review and approval timelines for each country. This would serve to inform further adaptations needed to current processes or the need for different/ new solutions.

## Conclusions

Data for more than 145,000 PACs across 156 countries collected for the first time by 18 global pharmaceutical companies for a 3-year period (2019–2021) illustrates how global regulatory complexity for PACs drastically impedes agile and timely implementation of changes, including those intended to continually improve products and processes and reduce the risk of drug shortages. Alleviating this complex problem requires better collaboration between industry and NRAs at a global scale to facilitate timely implementation of PACs.

The data presented show that only one country met the timeline of PAC approval within 6 months (inspired by the WHO recommended review timeline of 6 months for major changes) for all PACs that are submitted. 33 (22%) countries had more than half of the PACs taking > 6 months for approval, some up to more than 5 years. Many NRAs are doing a fairly good job of meeting the PAC CMC pre-approval time frame; however, because these PACs affect product going to multiple countries, the problem isn't solved until all affected countries approve the PAC. Thus the ‘tail end’ NRAs that take much longer for PAC review, negatively impact everyone.

Although the global PAC regulatory complexity problem has been known for more than 20 years, solutions introduced so far have treated the problem in ways that cannot solve complex problems. Taking a systems approach is needed to alleviate this complex problem and its impact.

8 approaches to optimize the Global PAC Management System, are described in this article. The 8 approaches include managing more PACs in the PQS only, using the PIC/S Recommendation Paper to assess PQS effectiveness during inspections, more regulatory reliance, and more global harmonization and standardization of how PACs are assessed. Each of the 8 approaches will help alleviate the complex PAC problem and collectively, they have the potential to significantly improve the situation. Only when all key stakeholders look beyond their operational boundaries will we collectively improve the Global PAC Management System and be able to drastically reduce this more than 2-decade-old complex problem.

## Data Availability

Thank you to the following companies for providing the data published in this paper: Amgen, Astellas, AstraZeneca, Bayer, Biogen, Boehringer Ingelheim, Bristol-Myers Squibb, CSL Behring, Gilead, Johnson & Johnson, Merck Sharp & Dohme Corp, Novartis, Novo Nordisk, Pfizer, Roche/Genentech, Sanofi, Takeda, Teva

## Footnotes

1 Deming’s 14 Points for Total Quality Management (TQM) is a widely recognized set of management practices to help companies increase their quality and productivity.
